# Perturbation of Parentally Biased Gene Expression during Interspecific Hybridization

**DOI:** 10.1371/journal.pone.0117293

**Published:** 2015-02-26

**Authors:** Diana Burkart-Waco, Kathie Ngo, Meric Lieberman, Luca Comai

**Affiliations:** The Genome Center and Section of Plant Biology, University of California Davis, Davis, California, United States of America; Universidad Miguel Hernández de Elche, SPAIN

## Abstract

Interspecific hybridization often induces epigenetic remodeling that leads to transposon activation, gene expression changes, and loss of imprinting. These genomic changes can be deleterious and contribute to postzygotic hybrid incompatibility. In *Arabidopsis*, loss of genomic imprinting of *PHERES1* and presumed failure of Polycomb Repressive Complex contributes to seed inviability observed in *A*. *thaliana X A*. *arenosa* interspecific hybrids. We used this species pair to further analyze the relationship between parentally biased gene expression and postzygotic hybrid incompatibility using two *A*. *thaliana* accessions, Col-0 and C24, with differential seed survival. We found that parentally biased expression was perturbed to a similar degree in both *A*. *thaliana* hybrids for *PHERES1*, *HDG3*, and six other normally paternally expressed genes. We propose that early genome remodeling and loss of imprinting of seed development genes induces lethality in both compatible and incompatible hybrids.

## INTRODUCTION

Sexual reproduction in plants results in the formation of two zygotes, the embryo and the endosperm, which together with maternal tissue form the seed. Sexual incompatibility between individuals of different species is commonly manifested by seed death, which is thought to depend on endosperm failure because the embryo can be rescued after microdissection [1]. Proper endosperm development in most angiosperms requires a 2:1 maternal:paternal contribution entailing multiple differentially contributed factors [2] including those resulting from genomic imprinting. Imprinting, the parent-of-origin dependent regulation of genes, ensues when one allele is preferentially expressed while the other allele is preferentially repressed. Expression bias manifested as down-regulation or complete suppression is associated with differential DNA methylation established by the coordinated action of methylating and demethylating pathways [3–6] such as is the case with imprinted gene *MEDEA* [7]. Differentially Methylated Regions (DMR) depending on parent of origin can be found in endosperm nuclear DNA, but not in embryo DNA, and are associated with genes that are preferentially expressed according to parent of origin. Another epigenetic pathway contributing to imprinting involves Polycomb Repressive Complex 2 (PRC2) and is exemplified by regulation of *PHERES1* [8,9], a paternally expressed gene (PEG) [10], whose suppression in the maternally-inherited allele requires FIS2, a PRC2 subunit protein. This regulation is likely to encompass multiple other PEGs [11,12]. At the same time, PRC2 contributes to regulation of maternally expressed genes, MEGs [10], exemplified by its action on the gene *MEDEA*, itself encoding a PRC2 subunit. Criteria to identify imprinted genes rely on expression and chromatin state [4,10–12]. In *A*. *thaliana*, about 300 genes fit at least some of these criteria. A majority are maternally expressed and most are regulated in the endosperm [10,12]. The observation of imprinted genes in endosperm is consistent with prevalence of imprinting in organs, such as the placenta, that nourish the embryo in mammals (reviewed in [13–15]). On the other hand, while embryonic transcription has been reported to be biparental from early seed development [10,16], a temporal gradient involving paternal delay has also been detected [17–19]. Indeed, a few genes are known to be imprinted in the embryo and developed sporophyte [11,20,21]. Therefore, imprinting can evolve in non-nutritive tissues albeit at a low frequency.

Endosperm-dependent seed failure in interspecific crosses has fostered the hypothesis that parental conflict drove the evolution of imprinting as a means of balancing opposing interests over the allocation of resources to progeny [22]. Inconsistencies between predictions of the parental conflict hypothesis and experimental data have stimulated alternative hypotheses [23,24]. For example, Beaudet and Jiang [25] proposed that haploid advantage and selection for hypervariability at dosage-sensitive loci may drive rapid evolution of imprinting. Whatever the explanation, plants display considerable variation in gene imprinting: rice and Arabidopsis share few imprinted genes [20] and substantial divergence in imprinting is obvious between rice and maize [26]. Rapid evolution of imprinting programs is likely to cause incompatibility in crosses between divergent individuals, resulting in failure of the endosperm. Consistent with this possibility, incompatibility between wild rice species and between *Arabidopsis thaliana* (At) and *Arabidopsis arenosa* (Aa) has been associated with subversion of imprinted gene regulation. In the latter system, seed death was associated with regulatory failure of two imprinted genes. Josefsson *et al*. [27] found that *PHERES1* (*PHE1*) misregulation is partially responsible for seed abortion in this interspecific cross as *PHE1* is biparental in hybrid crosses and knocking out *PHE1* significantly improved seed survival. Because *PHE1* is regulated by both the Polycomb Repressive Complex 2 and *DNA METHLYTRANSFERASE 1* (*MET1*), the aberrant transcription could result from either MET1 or PRC2 failure [27,28]. In the same experiment, the maternally expressed gene (MEG) *MEDEA* (*MEA*) displayed partial paternal expression. Following these findings, we set out to determine if there is a global loss of parentally biased gene expression in Arabidopsis wide-hybrids or if loss of imprinting is restricted to *PHE1* and *MEA*. We focused on two accessions of *A*. *thaliana*, Columbia (Col-0) and C24 (C24), which are known to display wide variation in hybridization success to *A*. *arenosa* (Aa) [29]. Additionally, we wanted to determine if differential survival of Col-0 X *A*. *arenosa* (0%) and C24 X *A*. *arenosa* (~17%) is the result of aberrant parentally biased gene expression. The expectation is that, if loss of imprinting underlies differential seed survival, there should be more severe parentally biased gene expression defects in incompatible Col-0 X *A*. *arenosa* hybrids.

To identify aberrant gene expression, we sequenced RNA and used single nucleotide polymorphism (SNP) analysis to characterize the parent-of-origin of transcripts of intraspecific reciprocal crosses of accessions Col-0 and C24, and interspecific crosses of the same two accessions to *A*. *arenosa*, a close sister species of *A*. *thaliana*. We found that in Arabidopsis wide-hybrids RNAs of most PEGs shifted from paternally biased to maternal. In addition to the previously reported *PHE1*, PEGs such as *HOMEODOMAIN GLABROUS* 3 (*HDG3*), *ALPHA-FUCOSIDASE* 1 (*ATFX1*), *ADMETOS* (*ADM*), displayed derepression of the maternal allele. At the same time more than 70 genes displayed unexpected paternal expression because they are not classified as PEGs in *A*. *thaliana* control intraspecific hybrids or in the literature. We consider alternative explanations for the general derepression of paternally biased, maternally imprinted genes and activation of novel paternal genes.

## MATERIALS AND METHODS

### Growth conditions

Wild-type Col-0 (CS6673) and C24 (CS22620), as well as male sterile lines that were hemizygous for a male sterility construct [30], were grown in 16 hours of light at 21°C and 8 hours of dark at 18°C. The male-sterile *A*. *thaliana* lines (ColA9 and C24A9) were either pollinated by *A*. *arenosa Strecno* (from M. Lysak and M. Koch) or wild-type Col-0 or C24. Several types of crosses with two biological replicates for each condition were used in these experiments: Intraspecific hybrids ColA9 X C24 and C24A9 X Col-0 and interspecific hybrids ColA9 X Aa and C24A9 X Aa plus *A*. *arenosa* controls [31]. No emasculation was needed because all egg donors either had a sterility construct or were self-incompatible (*A*. *arenosa*) [32,33].

### Plant material and RNA sequencing

For a detailed description of plant material and RNA sequencing see Burkart-Waco *et al*. (2013) [31]. At 3 days after pollination, fresh siliques were harvested (~50–80 siliques per replicate) and seeds were dissected from each silique and frozen on dry ice for RNA isolation using Plant RNA Reagent (Invitrogen, Carlsbad, California). All RNA was harvested from seed tissue. Libraries were constructed using homemade version of the Illumina RNA-Seq kit, using 10 μg of poly-A containing total RNA. After mRNA was isolated using poly-A purification, cDNA was synthesized and adaptors were ligated (S1 Table) in preparation for enrichment (12 cycles, Thermo Scientific Phusion High-Fidelity DNA Polymerase) and sequencing (Illumina GAIIx, 80 b paired end reads).

To obtain greater sequence depth of low- to moderate-abundance RNA, such as PEGs, we treated replicate 2 of Col-0 X C24 and C24 X Col-0 with the crab duplex-specific nuclease (see for rationale and methods [34]) and reamplified with 8–10 cycles of PCR prior to Illumina sequencing.

For independent validation of candidate MEGs and PEGs, three crosses of ColA9 X C24 and two crosses of ColA9 X Aa were performed as described above. Seed from approximately 40 siliques were harvested for each condition and RNA was isolated with Plant RNA Reagent. Approximately 1 μg of total RNA was obtained and treated with DNase 1 (New England Biolabs), according to manufacturer’s specifications. After RNAse treatment, cDNA was obtained with SuperScript III (Life Technologies). Select genes were amplified (see S2 Table for gene name and primer sequence) and pooled in equal quantities to 500 ng total using SYBR Green1 dye. After pooling, adaptors (S1 Table) were ligated with NEB Quick Ligase. Both biological replicates of Col-0 X Aa were technically replicated to ensure reproducibility of library preparation. KAPA Library Quant Kit (KAPA Biosystems) was then used to assess ligation efficiency and sequencing potential. PCR products were then sequenced on Illumina HiSeq 2000 (100 b paired end reads).

### Computational analysis

#### Sequence Preprocessing

Sequences were divided according to barcodes (S1 Table) and barcodes were removed. The Illumina 1.5+ format (fastq) reads were trimmed for adaptor contamination and bases with a quality score lower than Phred 20 were also excluded using a custom Python script. Reads that were shorter than 27 bases (for 80 b paired end) or 39 bases (for 100 b paired end). The Illumina quality scores were converted to Sanger scores, which are compatible with most aligners. Biological replicates were pooled for greater sequence depth. All sequence data for intraspecific control crosses and validation experiment are available for download at GEO (ID GSE56675) and for interspecific hybrids and *A*. *arenosa* (GSE42957).

Processed and pooled sequences were aligned to *A*. *thaliana* TAIR10 cDNA all gene models (available for download at ftp://ftp.arabidopsis.org//home/tair/Sequences//blast_datasets/TAIR10_blastsets/TAIR10_cdna_20101214_updated) using Burrows-Wheeler Aligner (BWA) version 0.5.8c [35]. Default settings were used with the addition of a trim quality of 20. SAM files and parsed pileups were generated using Samtools version 0.1.7 [36] as well as custom Python scripts.

#### SNP detection

For an overview of parent-of-origin expression detection, see S1 Fig. To detect parentally biased gene expression in *Arabidopsis* hybrids, we first sequenced Col-0, C24, and *A*. *arenosa* RNA using the Illumina sequencing platform to identify high-probability parent-specific SNPs. We removed reads with the same start position to ensure SNP coverage was not inflated. Because *A*. *arenosa* is an obligate outcrosser and displays frequent heterozygosity [33], we were concerned about ambiguity in SNP analysis. For SNP identification, we selected positions in all controls where 95% of reads contained a SNP at a coverage greater or equal to five reads. The mean percent SNP represents the sum of all positions within a single gene where a SNP was identified divided by the number of SNP identified for each gene; the mean coverage represents the sum of reads across all positions for which a SNP was observed divided by the number of positions. In RNA extracted from C24 seed derived by selfed crosses, we identified 84,255 SNP (mean percent SNP = 99.9% ± S.D. 0.5% and mean coverage = 27 reads ± S.D. 23 reads), which covered 13,376 genes (S1 File). In *A*. *arenosa* RNA, we identified 340,813 SNPs (mean percent SNP = 99.9% ± S.D. 0.4% and mean coverage = 16 reads ± S.D. 16 reads) covering 16,476 *A*. *arenosa* genes (S2 File). We then filtered out positions that had the same SNP base in both C24 and Aa. This provided us with 329,858 *A*. *arenosa* SNPs mapping to 16,422 genes (S2 Fig.). Approximately 15K Col-0 SNPs were also identified and removed from further analysis. Reciprocal control crosses and interspecific hybrids were then aligned to TAIR10 cDNA and SNPs identified in a manner similar to that used in the parents (coverage ≥ five reads with any SNP percentage).

#### Identification of parent of origin

Chi-square analysis using JMP 10.0 (SAS Institute) was used to identify differential maternal and paternal contributions for the following comparisons: 1) Col-0 X A. arenosa versus C24 X A. arenosa, 2) Col-0 X A. arenosa versus Col-0 X C24, 3) Col-0 X C24 versus C24 X Col-0, and 4) C24 X A. arenosa versus C24 X Col-0. First, the mean percent SNP and mean coverage for each library was calculated gene-by-gene. Then, for each gene, the approximate number of maternal reads or paternal reads was calculated using the following formulas:

$$N_{m}=C*\frac{P_{m}}{100}$$

$$N_{p}=C*\frac{P_{p}}{100}$$

Where N_m_ and N_p_ are the number of maternal or paternal reads, respectively. C is the total read count. P_m_ and P_p_ are the maternal or paternal allelic fraction, respectively, expressed as %.

To avoid division by zero, pseudocounts (adding one to the coverage for each library) were employed. Chi-square was determined in JMP and *P*-values were ranked and adjusted following the Benjamini-Hochberg correction [37]. Genes that in a pairwise comparison exceeded the allelic ratio of 2m:1p (*Padj* < 0.05) were considered MEGs. Genes that were below the allelic ratio of 1m:1p (*Padj* < 0.05) were considered to be PEGs. Both were reported in S3–S10 Files. Additional comparisons between hybrids and controls were also conducted using Chi-squared tests. Biological significance of outliers was assessed using “The Database for Annotation, Visualization and Integrated Discovery” (DAVID) [38]. Genes with known parental biases (from literature or The Arabidopsis Information Resource, TAIR) were used to confirm whether differential gene expression between hybrids and controls could be attributed to loss of imprinting.

#### Read counts

Read were mapped to TAIR10 cDNA (all gene models) as described above. Normalized read counts (normalized to smallest library) were generated using R package DESeq [39].

### Identification of a high reliability set of endosperm specific genes

We used an available dataset of tissue specific expression during seed development for 23,592 genes (Harada-Goldberg LCM dataset [40]). We focused on the preglobular and globular stages, which correspond to the timing of our analysis. For each gene, we derived a maximum value for all endosperm expression values and another maximum for the non-endosperm tissues (seed coat, suspensor, embryo). A log ratio of the maxima was calculated by the following formula:

$$log\frac{max\_endosperm\_value-2.3}{max\_nonendosperm\_value-2.3}$$

where 2.3 is a minimum threshold of expression that can be taken as the 0 in the LCM dataset. A log sum of the expression was by the formula:

log(endosperm_value+nonendosperm_value).

Low expressors were filtered by taking a minimum log sum of 1.8 resulting in 15,352 well expressed genes. For this set, we defined the top outliers of the distribution of the log ratio of the maxima using a Tukey outlier box plot where outliers (i.e. genes displaying high expression in endosperm and low expression in seed coat and embryo) are defined as those that exceed the 3rd quartile + 1.5*(interquartile range) of the distribution. This resulted in 940 genes (S11 File).

## RESULTS

### Parental bias in the interspecific transcriptome

**Fig 1:**
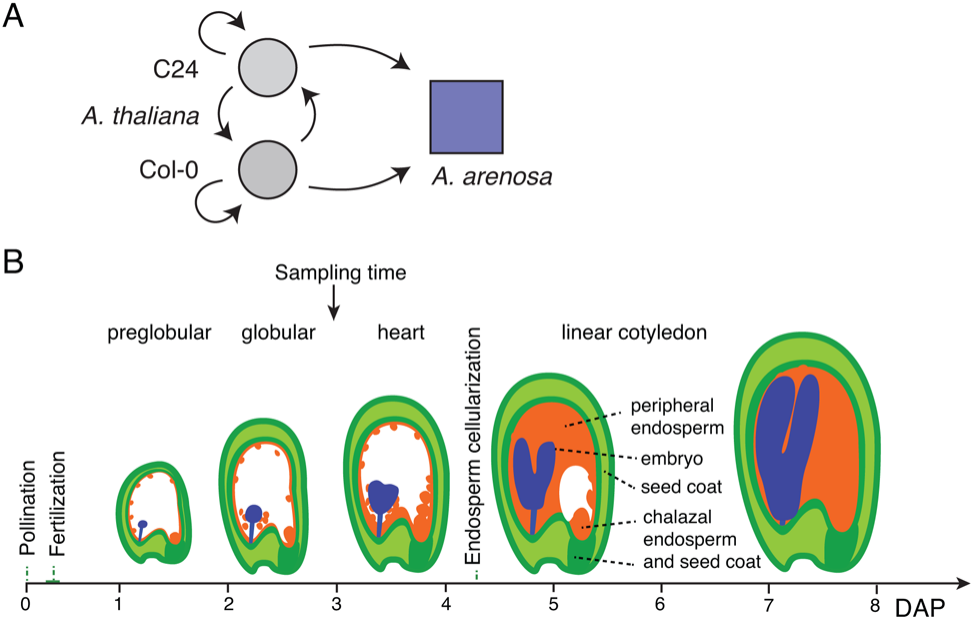
Hybrid crossing scheme and RNA sampling. **A**. Crosses between *A*. *thaliana* accessions, *A*. *thaliana* by *A*. *arenosa* interspecific crosses, and selfed crosses of parents were performed to detect parentally biased gene expression at 3 days after pollination. Arrows point to the males. **B**. Diagram of early *A*. *thaliana* development displaying seed compartment and stage of RNA sampling.

We selected two *A*. *thaliana* accessions, C24 and Col-0, for their differential compatibility, respectively, 17% and 0% live seed, when hybridized to the tester, *A*. *arenosa* accession Strecno (Fig. 1-A). To investigate whether parental expression bias contributed to incompatibility we compared early seed (Fig. 1-B) transcriptomes at 3 day after pollination (DAP) from the two hybrid crosses [31] hereby referred to as the “hybrids”, as well as reciprocal intraspecific crosses between *A*. *thaliana* Col-0 and C24, referred to as the “controls”. The *A*. *arenosa* genome displays ~5% nucleotide coding region divergence from the well characterized *A*. *thaliana* genome. We elected to map sequenced cDNA to the *A*. *thaliana* reference genes using an optimized C24 (S1 File, SNPs derived from C24 mRNA-seq libraries [31]) and *A*. *arenosa* (S2 File, SNPs derived from *A*. *arenosa* accessions Care and Strecno) SNP set to score allelic contribution (S1A Fig.). Control mappings indicated that this entailed an acceptable technical bias: genomic DNA and leaf cDNA from the natural allopolyploid (functionally a hybrid) *Arabidopsis suecica* representing 50:50 (*thaliana*:*arenosa*) sequence mixes yielded a ~60% *thaliana* SNP bias (see Methods for details). We set different SNP thresholds for categories of parental gene expression in intra- and interspecific hybrids: maternal at > 66% reflecting the expectation of 2:1 maternal:paternal genome ratio in endosperm, and paternal arbitrarily at > 50% SNP (both at Chi-square *Padj* < *0.05*; see methods for further explanations) (S1B Fig.). A similar method was also used by Wolff *et al*. to test allele-specific expression [12].

In the intraspecific control crosses we could determine parental origin for the mRNAs of ~10,800 genes (Table 1, S2 Fig., and S3–S4, S7–S8 Files). About 49% of the genes had a maternal SNP pattern, 50% were biparental, and 1% were paternal (Col-0 X C24, Table 1). This is consistent with seed composition during early development, which is biased toward maternal tissue and, to a lesser amount, endosperm. The trend found in control crosses was magnified in interspecific crosses where out of ~15,000 genes whose mRNAs could be assigned to parental genomes (S2 Fig. and S5–S6, S9–S10 Files), ~66% were classified as maternal, ~33% biparental, and less than 1% were paternal (Table 1).

**Table 1:** Parentally biased expression of 3 DAP Arabidopsis.

Cross ^a^	Total genes with SNPs ^b^	Expression Class	Informative Genes	Distribution of Reads
Col-0 X C24	10,754	Maternal	5,311	49.3%
		Biparental	5,365	49.9%
		Paternal	78	0.7%
C24 X Col-0	10,853	Maternal	4,350	40.1%
		Biparental	6,470	59.6%
		Paternal	33	0.3%
Col-0 X Aa ^b^	14,578	Maternal	10,899	74.8%
		Biparental	3,598	24.7%
		Paternal	80	0.5%
C24 X Aa	14,417	Maternal	9,762	67.7%
		Biparental	4,568	31.7%
		Paternal	87	0.6%

^a^ Col-0, *A*. *thaliana* accession Col-0; C24, *A*. *thaliana* accession C24; Aa, *Arabidopsis arenosa*

^b^ Single nucleotide polymorphism identified from alignments of sequence reads derived from *A*. *thaliana* C24 or from *A*. *arenosa* to TAIR10 cDNA (http://www.arabidopsis.org/)

**Fig 2:**
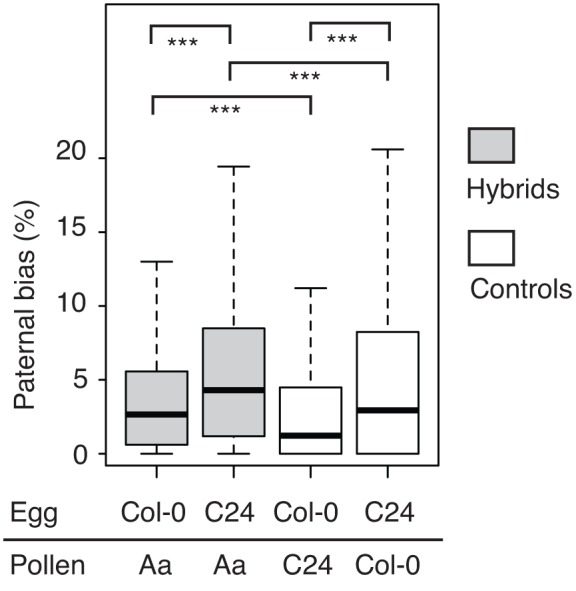
Overall changes in parental bias. Parental bias in transcripts of genes that were expressed in all crosses. Box plots illustrate how among commonly expressed genes interspecific crosses do not result in an overall erosion of paternal bias. Brackets indicate the outcome of Wilcoxon signed rank test (***: *P* < 0.001).

To compare patterns of parental bias across all experiments we selected 7,198 genes that were represented by a mean of five or more reads in each cross. Using this gene set, the mean paternal bias was slightly higher in the hybrids (4.18 and 6.04 for Col-0 x Aa and C24 x Aa, respectively) compared to controls (3.55 and 6.01 for Col-0 x C24 and C24 x Col-0, respectively), more markedly when C24 was the maternal parent (Fig. 2). While all means differed significantly according to Wilcoxon signed rank test, the differences were small. We concluded that overall paternal bias was slightly increased in the hybrids, but not grossly altered.

### Natural variation of parental regulation is mainly evident in the pollen parent

We focused on candidate MEGs and PEGs identified in control and hybrid crosses (thereafter in this section referred to as MEGs and PEGs) to determine how parental genotype affected their behavior. In the control crosses, Col-0 X C24 and C24 X Col-0, over 2000 genes with parental bias displayed accession-dependent effects (S3 Fig.). For example, of the 5,311 MEGs in Col-0 X C24, only two-thirds were classified as MEGs in C24 X Col-0 (S3A Fig.). Of the 78 PEGs in Col-0 X C24, only 11 were scored as PEGs in C24 X Col-0, with an additional 74 unique PEGs in C24 X Col-0 (S3B Fig.). In Col-0 X Aa, 51 of 80 PEGs (64%) were scored as PEGs in C24 X Aa (S3B Fig.).

Because a number of genes displayed accession-dependent effects, we set out to determine if parentally biased gene expression was preserved across species barriers. Our findings are presented in S4 Fig. The majority of candidate MEGs identified in the intraspecific control crosses were also maternal in the interspecific comparison (S4A Fig.). The 433 MEGs unique to Col-0 X C24 were enriched for cell killing (*Padj* < 0.05), but only relative to seed genes [31], not TAIR10 (S3 Table). A similar number of MEGs were identified in C24 X Col-0 versus C24 X *A*. *arenosa*, with no significant gene ontology enrichment, perhaps due to the small number of unique MEGs. For hybrid crosses, 6,021 genes were classified as candidate MEGs in Col-0 X Aa, but not Col-0 X C24, including six AGAMOUS-LIKE proteins (*AGL5*, *13*, *27*, *30*, *31*, *32*), known MEGs TRANSPARENT TESTA protein 1 and 2, and many other genes relating to intracellular transport (S3 Table). In C24 X Aa, we also identified *TTG1* and *AGL13* as MEGs.

We were concerned that the sets of candidate maternally-expressed genes were biased for maternally-derived seed coat proteins and that these genes were impairing our ability to detect differences between accessions. After filtering for endosperm specificity (see Methods, S11 File) [40], we defined approximately 200 candidate MEGs for each cross type, with greater than 50% of MEGs shared between hybrids and controls of the same maternal accession (S4A Fig.).

**Fig 3:**
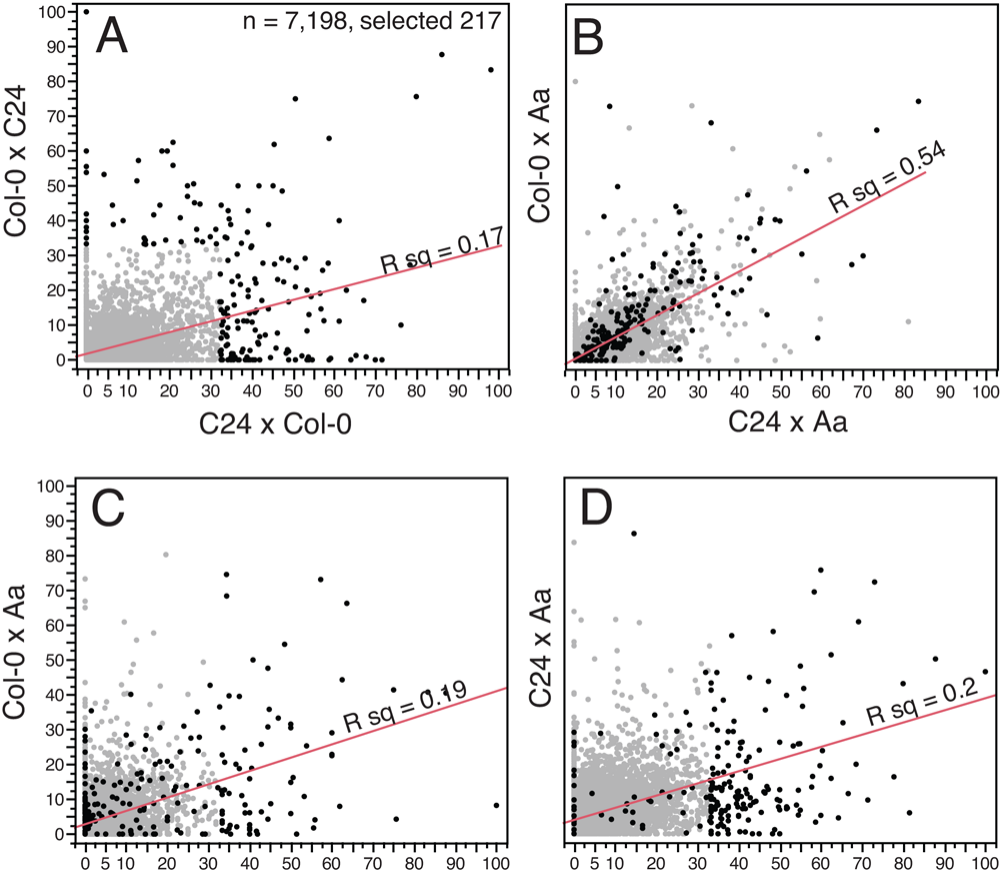
Paternal bias is most correlated in crosses sharing the same pollen parent. Fraction of paternal transcript for each gene crossing a threshold of minimum mean expression in all of the four studied crosses. Most genes are significantly parentally biased in at least one cross. The linear regression lines are shown. **A**. Paternal bias is poorly correlated in reciprocal intraspecific crosses of *A*. *thaliana* accessions Col and C24. Genes that exceed 33% paternal bias are highlighted in black and their position can be compared in plot B, C and D. **B**. Higher correlation in interspecific hybrids formed by pollination of *A*. *thaliana* by the same accession of *A*. *arenosa*. **C,D**. Sharing the same seed parent does not increase correlation of paternal bias.

PEGs displayed differential regulation in the intraspecific comparison. Less than 5% of all PEGs in both Col-0 and C24 hybrid were identified as PEGs in intraspecific control crosses (S4B Fig.). Since the PEGs in the above analysis displayed accession-dependent parental bias, we wanted to extend the analysis to a larger gene set and used the 7,198 genes defined above that are expressed in both control and hybrid crosses. Intraspecific and interspecific crosses share the same maternal seed parent: Col-0 and C24. They differ, however, in type and number of paternal seed parents: intraspecific crosses use two different parents, while interspecific crosses use a common pollen seed parent. The effect of divergent versus identical pollen seed parent can thus be compared and, as evidenced in Fig. 3, a better correlation of paternal bias is seen in the latter (R squared 0.54 vs 0.17, *P* < 0.001).

In summary, comparison of Col-0 and C24 reciprocal crosses revealed that PEG expression was not conserved (S4 Fig. and Fig. 3). Substantial conservation, however, was evident when the two accessions of *A*. *thaliana* were used as seed parent and mated to the same pollen parent, even if the latter belonged to a different species. Taken together these results suggested that paternal expression was largely dependent on the pollen parent. This is consistent with variation described when comparing imprinted gene identified with different combination of *A*. *thaliana* accessions [12]. We decided to focus on PEGs, which should not be biased by maternally-derived tissue contamination.

### The fate of PEGs during interspecific hybridization

To understand the status of paternal gene expression post-hybridization, a literature search was performed to identify previously characterized imprinted genes. We found references to 200 MEGs and PEGs, with approximately 65 genes with predicted paternal biased expression in the endosperm and embryo [3,7,10–12,16,41–44] (S4 Table). Using alignments to *A*. *thaliana* cDNA and data from our *de novo* SNP analysis, we were able to determine that some of these genes were expressed in hybrid and control crosses (S4 Table). Since reciprocal interspecific crosses were not possible because of unilateral incompatibility between *A*. *thaliana* and *A*. *arenosa* [32], our analysis was limited to comparing parental ratios between interspecific hybrid and corresponding intraspecific controls (i.e. Col-0 X C24 versus Col-0 X *A*. *arenosa*).

**Fig 4:**
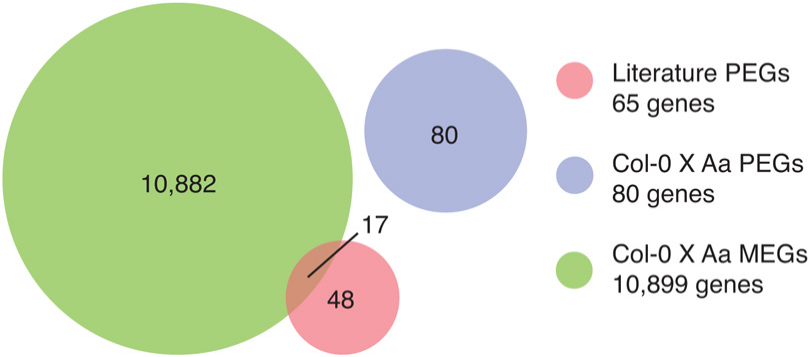
Interspecific paternally expressed genes vary from known imprinted genes. Candidate maternally and paternally expressed genes identified in Col-0 X *A*. *arenosa* were compared to paternally expressed genes identified in previous parent-of-origin studies. The number of candidate genes for each classification is given. Paternally expressed genes in Col-0 X *A*. *arenosa* crosses display no overlap with literature genes. This class of genes was designated as *de novo* PEGs in interspecific crosses. In addition, 17 known paternally expressed genes were identified as candidate maternally expressed genes in Col-0 X *A*. *arenosa*.

The analysis of paternally-biased genes confirmed the disruption of paternal expression observed in the overall analysis above. Of 65 previously described PEGs (S12 File), 52 were expressed with sufficient coverage for SNP detection. None were paternally biased in Col-0 X Aa (Table 2, Fig. 4, and S9 and S12 Files). We observed maternal bias for 17 genes (Table 2, Fig. 4, and S5 File) including *SU(VAR)3–9 HOMOLOG 9* (*SUVH9*), *ATFXG1*, *CHROMATIN REMODELING 34*. In the case of characterized PEG *PHERES1*, expression was entirely dependent on the maternal allele as all reads matched the Col-0 sequence perfectly (Table 2, S5 Fig., and S13 File).

**Table 2:** Abnormal Paternally Expressed Genes (PEGs).

			Col-0 X Aa		C24 X Aa		Col-0 X C24		C24 X Col-0	
Gene	Gene product	Aa expr. ^a^	Pat % ^b^	N^c^	Pat %	N	Pat %	N	Pat %	N
*AT1G57800*	VIM5	traces	-	607	-	728	96.3^d^	58	73.7	145
*AT1G65330*	PHERES1	none	-	802	-	747	95.6^d^	407	98.1^d^	202
*AT1G67820*	phosphatase	traces	40.4	27	50.0	56	87.7^d^	88	87.9^d^	149
*AT1G67830*	ATFXG1	Low	4.3	1083	6.0	1319	-	53	81.6^d^	188
*AT2G32370*	HDG3	none	-	234	-	281	87.9^d^	54	54.2	128
*AT2G40520*	nucleotidy-ltransferase	none	-	49	-	42	-	546	89.0	303
*AT3G49770*	unknown	none	-	4	-	27	100.0^d^	23	77.5	267
*AT3G50720*	kinase	none	-	3	-	27	100.0^d^	34	95.0^d^	48
*AT4G31900*	PKR2	none	-	42	-	197	100.0	133	84.4	166

^a^ Expression in *Arabidopsis arenosa* sib-cross as evaluated from aligned reads on IGV browser [49].

^b^ Mean % of paternal SNP (mean coverage = > 5 reads per gene).

^c^ Coverage determined by mean number of reads that mapped to TAIR10 (average of two biological replicates).

All reads were normalized to library with R package DESeq.

^d^Genes expressed with a significant *paternal* bias (Chi-square test, *Padj* < 0.05).

-, no SNPs detected.

We set out to validate the observed loss of paternity with additional independent replicates from 3 DAP seed. Our findings are presented in Table 3. We identified 11 previously described PEGs that were PEGs or biparental in Col-0 intraspecific crosses, but we could only identify SNPs between Col-0 and C24 for eight of these genes. Of the genes with diagnostic SNPs, 8/8 were confirmed to be paternal with > 63% C24 SNP in Col-0 X C24 hybrids (*ADM*, *PHE1*, and *ATFXG1* to name a few). In contrast, these genes manifested a maternal footprint in Col-0 interspecific hybrids at 3 DAP. Of the 7/11 PEGs with *A*. *arenosa* SNPs, 5/7 were maternal (<15% *A*. *arenosa* allele), including *PHE1*, *ATFXG1*, and *SUVH7*. *AT2G40520* and *YUCCA10* were biparental in Col-0 hybrid, paternal expression with an average of ~71% *A*. *arenosa* across nine parent-specific SNPs. The remaining four genes had sufficient read coverage for SNP detection, but we were unable to assign reads to either parent. Interestingly, most of these *A*. *thaliana* PEGs, with the exception of alpha-fucosidase *AtFXG1* (*AT1G67830*), displayed traces or no detectable expression in *A*. *arenosa x A*. *arenosa* crosses. The lack of expression is surprising since two of these genes, *PHERES1* and *ADMETOS*, have been attributed a function [8,45]. In the case of *PHERES1*, expression assays based on RT-PCR on *A*. *arenosa* seed cDNA with genome-specific primers failed to yield positive data in the time range tested (2–8 DAP, results not shown). It is thus possible that these proteins have no function in early seed development of *A*. *arenosa*.

**Table 3:** Validation of Paternally Expressed Genes (PEGs) misexpression.

Gene	Gene product	N SNP^a^ Col-0 X C24	Cov^b^ Col-0 X C24	%SNP^c^ Col-0 X C24	StDev^d^ Col-0 X C24	N SNP Col-0 X Aa	Cov Col-0 X Aa	%SNP Col-0 X Aa	StDev Col-0 X Aa
*AT1G17770*	SUVH7	0	9529^e^	NA	NA	3	57	5.79	0.8
*AT1G60410*	F-box	0	344^e^	NA	NA	8	156	13.97	2.6
*AT1G65330*	PHE1	2	24922	86.79	0.0	0	881^e^	NA	NA
*AT1G67820*	Phosphatase	6	7224	92.21	0.5	4	83	15.03	6.8
*AT1G67830*	ATFXG1	3	22030	83.15	0.4	8	173	11.47	3.7
*AT2G32370*	HDG3	1	13740	63.05	0.0	0	431^e^	NA	NA
*AT2G36560*	DNA-binding	3	51959	85.27	0.1	2	113	6.63	0.5
*AT2G40520*	Nucleotidyltransferase	3	2961	70.89	1.6	6	123	47.67	18.9
*AT4G11940*	ADM	4	42576	78.70	0.1	0	468^e^	NA	NA
*AT4G15400*	Acyl-transferase	0	39382^e^	NA	NA	0	612^e^	NA	NA
AT1G48910*	YUC10	1	1972	76.36	0.0	9	172	70.86	3.9

^a^ The number of SNPs (% paternal, either % C24 or % *A*. *arenosa*) identified in PCR amplicon during read alignment

^b^ Mean number of reads for all SNPs in each gene

^c^ Mean percent SNP per PCR amplicon

^d^ Standard deviation in percent SNP

^e^ Genes with no detectable SNP. Expression is listed as read count per locus.

*While this gene is identified as PEG in literature, it was not identified as PEG by our criteria. In addition, percent SNP detected in Col-0 X Aa was not significantly different from percent SNP in Col-0 X C24 (Chi-square analysis normalized to lowest coverage at P > 0.01).

### PEG candidates of *A*. *arenosa*

Our analysis found a category of potential PEGs that were unreported as such in *Arabidopsis thaliana* (Fig. 4, S4 Fig., S14 File). Failure to identify biased parental expression of these genes in the control crosses has at least three explanations. Firstly, these genes could be similarly biased in *A*. *thaliana* intraspecific crosses, but escaped detection because no SNPs were available to identify them. However, while most of these genes displayed SNP in C24, they were poorly or not expressed in our 3 DAP seed data. Second, they could be induced paternally in response to hybridization and represent a set of genes not normally expressed in seed, but sensitive to a hypothetical regulatory disruption. Third, their paternal expression could be specific to *A*. *arenosa*, but not *A*. *thaliana* seed development. Consistent with the last explanation, expression of these genes was low in *A*. *thaliana*, but much higher in *A*. *arenosa* x *A*. *arenosa* crosses (normalized mean read count *A*. *arenosa* = 2161 reads, S.D. = 4764; mean read count Col-0 X C24 = 59 reads, S.D. = 238 reads).

Closer examination of the PEG candidates was interesting. Categorical classification of the predicted protein products was consistent with that of genes expressed during seed development as they were enriched for cell wall modifying activities, secretion, and ribosome constituents. Of the 77, 62 were represented in the AtGenExpress Affymetrix array set and 40 had sufficient expression values to be assessed for endosperm-specific expression. By requiring high expression in endosperm and low expression in embryo and maternal tissue (see Methods), we defined 940 high-confidence endosperm specific genes in the Harada seed development set [40]. When this conservative set was compared to the *A*.*arenosa* PEG candidates, 13 were common to both sets (expected mean = 2.46, hypergeometric P = 4.12E-7), indicating by a stringent test that 32.5% of the PEG candidates (13/40) displayed endosperm-specific expression in *A*. *thaliana* (see sample in Table 4). An unusual regulatory pattern involved gene *AT5G59845*: expression of the paternal allele was associated with suppression of the maternal allele, which was well expressed in the controls. This response was unexpected because this gene, encoding a gibberellin responsive factor, is predominantly expressed in the seed coat in *A*. *thaliana* [40]. In the interspecific hybrid, however, this normally highly expressed gene was abated and the lower-level paternal transcript was predominant. Another regulatory pattern involved maintenance of biparental or paternal expression and is exemplified by gene *AT2G28680*, encoding a cupin family protein, which is expressed predominantly in the embryo suspensor and micropylar endosperm. It displayed a paternal or biparental pattern in all crosses. The most common regulatory pattern, however, involved strong induction in the hybrid compared to the *A*. *thaliana* parent, but moderate induction with respect to the *A*. *arenosa* parent.

**Table 4:** Exemplary genes uniquely paternal in the *A. thaliana x A. arenosa* cross.

Gene	Predicted product	Expression in At	Expression pattern	Paternal SNP mean % ^a^	
				Col-0 x Aa	C24 x Aa
*AT1G24520*	Pollen protein 1	Low	Flowers, early seed	96	87
*AT1G34460*	Cyclin B1	Low	Shoot apex, flower, seed	74^e^	86
*AT1G36240*	Ribosomal protein L7a	Low ^b^	Flower, seed ^b^	100	100
*AT2G01880*	Purple acid phosphatase	Low	Root, flower, early seed	92	91
*AT2G27120*	DNA Pol. Epsilon subunit	Low	Flower, seed	71^e^	84
*AT3G43800*	Glutathione S-transferense	Medium	Most organs, early seed	65^e^	75
*AT3G51420*	Strictosidine synthase-like 4	Low	Leaf, flowers, early seed	96	89
*AT4G35010*	Beta-galactosidase	High ^c^	Flowers, seed	100	100^e^
*AT5G42223*	Defensin-like protein	High ^d^	NA	88	66
*AT5G43640*	Ribosomal protein S19	Medium	Leaf, flower, seed	89	95
*AT5G48140*	Pectin lyase	Low	Flower, seed	96	100
*AT5G59845*	Gibberellin-regulated protein	High	Flower, seed	73	29^e^

^a^ Derived from selected, informative SNP detected with aligned reads visualized on IGV browser

^b^ Paralog *AT1G77940* is strongly expressed in most tissues; *AT1G36240* is significantly upregulated in Col-0 x Aa cross compared to Col-0 selfed crossed [31].

^c^ Based on AtgenExpress profile. Low in our data. Difference may be due to paralogs.

^d^ Significantly downregulated in Col-0 x Aa compared to selfed crossed Col-0 [31].

^e^ Not significantly (*P-adjusted* > 0.05) different from 50/50 maternal/paternal ratio.

In summary, our results reveal widespread changes in PEG regulation during wide hybridization, consistent with the earlier observation on *PHERES1* [27]. The early, large-scale PEG remodeling occurring at a critical time of seed development could be a major contributor to interspecific incompatibility.

## DISCUSSION

We examined the pattern of parentally regulated genes during interspecific hybridization. We compared two polymorphic *A*. *thaliana* accessions crossed to each other or to the species *A*. *arenosa*, a cross in which the chosen accessions display differential incompatibility. Candidate imprinted genes are detected by genotyping allelic parental contribution to the mRNA of progeny. In our case, the interspecific comparison was facilitated because polymorphisms are at least 10 times as frequent as in the intraspecific comparison between *A*. *thaliana* accessions. We chose to assay an early developmental stage, globular embryo at 3 DAP, because incompatibility manifests first at this time [31]. Each seed was sampled whole due to the challenge of separating the 90 to 120 endosperm nuclei from embryo and maternal seed integument tissue. Endosperm mRNA was thus obtained mixed with embryonic and maternal RNA. Studies employing torpedo-stage seed (7–8 DAP) [10,11], could more easily separate endosperm from maternal tissues. In our sampling, we expected that maternal contribution should appear enhanced and paternal contribution diluted for genes expressed in maternal tissue. Sampling of embryo mRNAs, which is currently believed to be maternally biased [17,18] or express a 50–50 mix of parental alleles [16], could also alter the measured parental bias. Nonetheless, mRNAs representing paternal alleles was readily identified by their divergence, and could only result from zygotic tissues. Large paternal bias mRNAs should most likely derive from endosperm because imprinting is very rare in embryos. As a result, we could ascertain the effect of interspecific parental bias for paternally expressed genes including characterized imprinted genes.

Our analysis revealed that paternally expressed genes were perturbed in response to hybridization, but that the degree of perturbation did not vary between crosses where the seed mother was incompatible accession Col-0 and compatible C24. In addition to *PHERES*1, at least nine known PEGs become maternally-biased during interspecific hybridization (Table 2 and Table 3). Increased maternal expression could derive from maternal tissue, which is the same as in the intraspecific crosses, or by expression of the maternal allele in the endosperm, a hypothesis which we favor because it is more parsimonious. Such conversion of PEGs into MEGs would be unexpected in the context of our current understanding of imprinting mechanisms. PEG regulation involves expression of the paternal allele with concurrent suppression of the maternal one. Suppression of the female allele is thought to be the result of an epigenetic state determined in the female lineage. Derepression of maternal alleles in these reverted loci indicates that this repressed epigenetic state is not hard set. Rather, post-fertilization factors provided directly or indirectly by the male must be required to either maintain or determine the state. Interestingly, expression in *A*. *arenosa* sib-crosses could not be documented for most of the *A*. *thaliana* known PEGs that were found reverted in this study. This could be a technical problem: for unknown reasons, seed and seedling stage RNA-seq libraries of *A*. *arenosa* are challenging to make and were produced only through multiple attempts. As a result, there was lower overall sequencing coverage in this genotype and several of these genes might be expressed at low level. In the case of *PHERES1*, however, reverse transcription PCR with *A*. *arenosa*-specific primers failed to demonstrate any expression during early *A*. *arenosa* seed development. At least in the case of this gene, it is thus possible that the factor required for maternal *PHERES1* suppression may be connected to the paternal *PHERES1* transcript itself. Increasing the ploidy of the seed parent *A*. *thaliana* relieves the maternal induction of *PHERES1* even though paternal *PHERES1* mRNA is not observed [27], indicating that absence of the cognate paternal mRNA is not sufficient for maternal allele derepression. Under incompatible conditions, therefore, a mechanism resulting in apparent overcompensation activates the maternal allele.

Our observations can be interpreted in two ways. According to the first explanation, it is possible that interspecific hybridization causes a systematic failure of PEG regulation leading to both the ectopic expression as PEGs of genes that are PEGs in either parental species, and to the conversion of most PEGs to MEGs. Many of the changes observed during interspecific hybridization are consistent with failure of the Polycomb Repressive Complex 2 (PRC2) [31], which plays an important role in the regulation of imprinting [8,9,46]. Failure of PRC2 could thus result in the syndrome described here.

A second, alternative and perhaps additive explanation is based on rapid evolution of imprinted gene regulation [26,47,48]. Three PEG-candidates behaved as MEGs in our control intraspecific crosses: *AT3G03980*, *AT5G39260*, and *AT5G59845*. While conversion of PEGs to MEGs was not widespread in crosses between *A*. *thaliana* accessions, it is plausible that further divergence between *A*. *thaliana* and *A*. *arenosa* might contribute to the frequent conversion observed in interspecific crosses. Further, Fig. 3-A illustrates the low correlation in paternal bias during reciprocal intraspecific crosses. Surprisingly, a better correlation is observed when different seed-parents are mated to the same pollen-parent of a different species (Fig. 3-B). This suggests an important role for pollen parents in determining PEGs. The eight failed PEGs, could thus be explained by the fact that these genes are not PEGs in *A*. *arenosa*.

Seventy-seven additional genes behaved as PEGs in interspecific hybridization, although they were not detectable as PEGs in *A*. *thaliana*. We define these as *A*. *arenosa* PEG candidates. Verification of their pattern of parental origin in *A*. *arenosa* x *A*. *arenosa* crosses was not attempted because this analysis is complicated by high heterozygosity in individuals of this species. The set of *A*. *arenosa* PEG candidates are enriched for endosperm genes, including genes predicted to encode proteins whose categorical classification is consistent with that of imprinted genes, being significantly enriched for proteins involved in cell wall metabolism and cell growth (S5 Table) [10]. Is the number of these potential PEGs too high, given that relatively few PEGs have been discovered in prior plant studies? Perhaps, PEG numbers have been underestimated. Under rapidly evolving imprinting [26,48], reciprocal crosses of two diverged accessions could fail to identify genes that are imprinted in one and not in the other. Under this scenario of rapid evolution, one might expect to find similar variation in MEGs. We did not focus on MEG regulation because of the presence of maternal tissue in our sample. Nevertheless, conversion of MEGs to PEGs would have been readily detected by the appearance of characteristically *A*. *arenosa* alleles and we can thus feel confident that such conversion was not common. The instability of PEG regulation could result from differences in the evolutionary speed of regulatory innovation between PEGs and MEGs.

In conclusion, the observed instability in paternally expressed genes during interspecific hybridization indicates either regulatory disruption or rapid evolution of imprinting. While this is likely to represent a major hybridization barrier between diverging populations, the different phenotype displayed by *A*. *thaliana* accessions Col-0 and C24 during hybridization with *A*. *arenosa* could not be explained by differences in imprinted gene regulation.

## Supporting Information

S1 Fig Experimental design: parent-of-origin expression detection. (A) High probability parent-specific single nucleotide polymorphisms (SNPs) (95% SNP at 5 read coverage) were identified for *A*. *thaliana* accession C24 (S1 File) and *A*. *arenosa* accession Strecno (S2 File) to determine parental contribution in intraspecific (Col-0 X C24 and C24 X Col-0) and interspecific (Col-0 X *A*. *arenosa* and C24 X *A*. *arenosa*) hybrids. (B) Genes that were expressed in both intraspecific and interspecific hybrids were classified based on paternal contribution (S3–S6 Files). Chi-square analysis in JMP was used to test if paternal contribution differed within species (Col-0 X C24 versus C24 X Col-0) and between species (Col-0 X C24 versus Col-0 X *A*. *arenosa*). (C) Paternal contributions in (B) were then tested against a set of genes with known paternal contributions.
(EPS)

S2 Fig Identification of parent-specific single nucleotide polymorphisms. Single nucleotide polymorphisms (SNPs) were detected relative to *A*. *thaliana* cDNA for C24 and *A*. *arenosa* (Aa) at 3 days after pollination. These SNPs were observed in intraspecific hybrid crosses (Col-0 X C24 and C24 X Col-0) and interspecific hybrid crosses (Col-0 X Aa and C24 X Aa). The number of genes with detectable SNPs at coverage of ≥ 5 reads per SNP are given by colored circles.
(EPS)

S3 Fig Analysis of accession-specific parental contributions. A Chi-square analysis of SNP ratio was used to classify genes parent-of-origin. Candidate maternally expressed genes (MEGs) exhibited more than 66% maternal contribution while paternally expressed genes (PEGs) manifested as more than 50% paternal (*Padj* < 0.05). The Venn diagrams above depict genes with differential paternal contributions from (A) mom and (B) dad between accessions Col-0 and C24 in intra- and interspecific crosses. Aa; *A*. *arenosa*.
(EPS)

S4 Fig Intra- and interspecific hybrids vary in paternal expressed genes. A) Comparison of maternally biased genes between intraspecific (either Col-0 X C24 or C24 X Col-0) and interspecific hybrids. The majority of genes that were identified as maternal in Col-0 X C24 were also expressed from *A*. *thaliana* Col-0 in the interspecific hybrid. B) Comparison of paternally biased genes (genes with < 50% SNP). While paternally expressed genes were identified in all crosses, there was little overlap between genotypes.
(EPS)

S5 Fig Identification of paternal single nucleotide polymorphisms in PHERES. To confirm the parent of origin of imprinted gene PHERES1 in interspecific hybrids, RNA-seq reads (red, forward read or blue, reverse read) from Col-0 X A. arenosa (Aa) were aligned to TAIR10 cDNA and visualized using Integrative Genomics Viewer. Vertical lines within reads indicate the presence of single nucleotide polymorphisms (from variation in A. arenosa or sequencing errors). No SNPs are apparent for PHERES1 (left). PHERES2 (right) is included as control to ensure that the above observed maternal gene expression of PHERES1 is not due to mismapping of paternal reads to a close homolog PHERES2.
(EPS)

S1 Table Adaptor and barcode sequences. (PDF)

S2 Table Primers for paternally expressed gene (PEG) validation. (PDF)

S3 Table Gene Ontology of differential parental contributions. (PDF)

S4 Table Expression analysis of imprinted genes in the literature. (PDF)

S5 Table Unique PEGs related to cell growth and expansion. (PDF)

S1 File C24_SNPs. C24 SNP set generated by aligning sequenced 3 DAP C24 to TAIR10.
(DAT)

S2 File Aa_SNPs. *A*. *arenosa* SNP set generated by aligning sequenced 3 DAP seed mRNA to TAIR10.
(DAT)

S3 File ColxC24_MEG. Identification of MEGs in Col-0 x C24.
(DAT)

S4 File C24xCol_MEG. Identification of MEGs in C24 x Col-0.
(DAT)

S5 File ColxAa_MEG. Identification of MEGs in Col-0 x Aa.
(DAT)

S6 File C24xAa_MEG. Identification of MEGs in C24 x Aa.
(DAT)

S7 File ColxC24_PEG. Identification of PEGs in Col-0 x C24.
(DAT)

S8 File C24xCol_PEG. Identification of PEGs in C24 x Col-0.
(DAT)

S9 File ColxAa_PEG. Identification of PEGs in Col-0 x Aa.
(DAT)

S10 File C24xAa_PEG. Identification of PEGs in C24 x Aa.
(DAT)

S11 File Endosperm_specific_genes. List of endosperm specific genes.
(DAT)

S12 File Lit_PEGs_List_Merged_SNPs. Literature PEGs merged with SNP data from intraspecific and interspecific hybrids.
(DAT)

S13 File PHERES_seq. *A*. *arenosa* sequence data for *PHERES1* and *PHERES2* homologs.
(TXT)

S14 File Denovo_PEG. *De novo* PEGs identified in Col-0 X *A*. *arenosa* crosses.
(DAT)
