# Supplementary material for: Perturbation of Parentally Biased Gene Expression during Interspecific Hybridization
Source: PLoS One. 2015 Feb 26;10(2):e0117293. doi: 10.1371/journal.pone.0117293 (PMC4342222; doi:10.1371/journal.pone.0117293)
Supplement: S1 Table — (PDF) [file pone.0117293.s006.pdf]

TABLE S1

Adaptor and barcode sequences

Genotype	Barcode name	Sequence[1]
**Adaptor sequences**		
**mRNA-seq**		
For ColA9 X Aa, C24A9 X Aa, ColA9 X Col-0, C24A9 X C24, and 2x-Aa X 2x-Aa see Burkart-Waco *et al.* 2013.		
ColA9 X C24 #1	>adA2_CTAGT	**CTAGT**AGATCGGAAGAGCGGTTCAGCAGGAATGCCGAG
ColA9 X C24 #1	>adB2_CTAGT	ACACTCTTTCCCTACACGACGCTCTTCCGATCT**ACTAG**T
ColA9 X C24 #2	>adA2_GACAC	**GACAC**AGATCGGAAGAGCGGTTCAGCAGGAATGCCGAG
ColA9 X C24 #2	>adB2_GACAC	ACACTCTTTCCCTACACGACGCTCTTCCGATCT**GTGTC**T
C24A9 X Col-0 #1	>adA2_TGCGT	**TGCGT**AGATCGGAAGAGCGGTTCAGCAGGAATGCCGAG
C24A9 X Col-0 #1	>adB2_TGCGT	ACACTCTTTCCCTACACGACGCTCTTCCGATCT**ACGCA**T
C24A9 X Col-0 #2	>adA2_AAGCG	**AAGCG**AGATCGGAAGAGCGGTTCAGCAGGAATGCCGAG
C24A9 X Col-0 #2	>adB2_AAGCG	ACACTCTTTCCCTACACGACGCTCTTCCGATCT**CGCTT**T
**Adaptor sequences**		
**Gene validation**		
ColA9 X Aa #1.1	>Bioo28A_CAAAAG	Available for order through http://www.biooscientific.com/
ColA9 X Aa #1.1	>Bioo28B_CAAAAG	Available for order through http://www.biooscientific.com/
ColA9 X Aa #1.2	>Bioo34A_CATGGC	Available for order through http://www.biooscientific.com/
ColA9 X Aa #1.2	>Bioo34B_CATGGC	Available for order through http://www.biooscientific.com/
ColA9 X Aa #2.1	>Bioo29A_CAACTA	Available for order through http://www.biooscientific.com/
ColA9 X Aa #2.1	>Bioo29B_CAACTA	Available for order through http://www.biooscientific.com/
ColA9 X Aa #2.2	>Bioo35A_CATTTT	Available for order through http://www.biooscientific.com/
ColA9 X Aa #2.2	>Bioo35B_CATTTT	Available for order through http://www.biooscientific.com/
ColA9 X C24 #1	>Bioo32A_CACTCA	Available for order through http://www.biooscientific.com/
ColA9 X C24 #1	>Bioo32B_CACTCA	Available for order through http://www.biooscientific.com/
ColA9 XC24 #2	>Bioo40A_CTCAGA	Available for order through http://www.biooscientific.com/
ColA9 X C24#2	>Bioo40B_CTCAGA	Available for order through http://www.biooscientific.com/
ColA9 X C24 #3	>Bioo41A_GCGCTA	Available for order through http://www.biooscientific.com/
ColA9 X C24 #3	>Bioo41B_GCGCTA	Available for order through http://www.biooscientific.com/

Adaptor A contains 5’ phosphate group
