# Supplementary material for: Perturbation of Parentally Biased Gene Expression during Interspecific Hybridization
Source: PLoS One. 2015 Feb 26;10(2):e0117293. doi: 10.1371/journal.pone.0117293 (PMC4342222; doi:10.1371/journal.pone.0117293)
Supplement: S2 Table — (PDF) [file pone.0117293.s007.pdf]

## TABLE S2

Primers for paternally expressed gene (PEG) validation

| Locus | Name | Predicted Expression | Primer Sequence | Product size (cDNA) | Amplifies |
|---|---|---|---|---|---|
| AT1G17770 | *SUVH7* | PEG | 5'-TGGACAGTGACAGGAAAATCC<br>3'-CACCGCACTCGTAAATCAAT | 433bp | Both |
| AT1G60410 | | PEG | 5'-TGTATTTTGAGGGGGTCAAGTT<br>3'-CCAATTCTATGTATTTTAAGGCTTTC | 268bp | Both |
| AT1G65330 | *PHERES1* | PEG | 5'-TAGCCCGTACAACTCGATCC<br>3'-CGGTTCTCATCACGTAGCTT | 183bp | Both |
| AT1G67820 | | PEG | 5'-GGAAGGGATGATGAAAAGGA<br>3'-GGGTGATTCATTCTCTGAGTTG | 417bp | Both |
| AT1G67830 | *AtFXG1* | PEG | 5'-TTGAAACCGAAGTTCCTGAGA<br>3'-CACAATCTTCTTCATCCCACA | 419bp | Both |
| AT1G48910 | *YUC10* | PEG | 5'-GTGGTGATTGTGGGAGCTG<br>3'-GATGTCGAAACGGGCAAC | 261bp | Both |
| AT2G32370 | *HDG3* | PEG | 5'-TCCTCCTAACACTGTCTTTGACT<br>3'-GTCAGGATCACCACCTCCAT | 277bp | Both |
| AT2G36560 | | PEG | 5'-AAATGGTTCTCGTGGGATCA<br>3'-AATGAGACTCCCAGCCAAAG | 250bp | Both |
| AT2G40520 | | PEG | 5'-CCGTATTCTCGGTGCCAAC<br>3'-GACAGGCTCCTCTACGTCTTG | 547bp | Both |
| AT4G11940 | | PEG | 5'-CCAATCCAACCATTAAGGAGA<br>3'-ACTCTGATCCAGGCAAATCA | 159bp | Both |
| AT4G15400 | *ABS1* | PEG | 5'-GGAGAGCGAGTTTGAGATCC<br>3'-CCATGCTTCTACACCATCACC | 316bp | Both |

Aa, *Arabidopsis arenosa*
At, *Arabidopsis thaliana*
