# Supplementary material for: Perturbation of Parentally Biased Gene Expression during Interspecific Hybridization
Source: PLoS One. 2015 Feb 26;10(2):e0117293. doi: 10.1371/journal.pone.0117293 (PMC4342222; doi:10.1371/journal.pone.0117293)
Supplement: S3 Table — (PDF) [file pone.0117293.s008.pdf]

TABLE S3

Gene Ontology of differential parental contributions

Parental Bias	Cross	GO Category[a]	No. of Genes[b]	Fold Enriched[c]	*P*-value[d]
MEG	Unique Col-0 X Aa	GO:0046907~intracellular transport	159	1.4	0.0003
		GO:0015031~protein transport	186	1.3	0.0005
		GO:0008104~protein localization	191	1.3	0.0117
		GO:0006412~translation	215	1.3	0.0125
		GO:0006886~intracellular protein transport	111	1.4	0.0293
	Unique Col-0 X C24	GO:0031640~killing of cells of another organism	12	5.8	0.0038

[a] GO Category, as indexed by DAVID (http://david.abcc.ncifcrf.gov/).
[b] Number of differentially expressed genes in GO category.
[c] Fold enrichment relative to expressed seed genes (see Burkart-Waco *et al.* 2013 for seed gene set).
[d] Benjamini-Hochberg corrected *P*-value.
