# Supplementary material for: Perturbation of Parentally Biased Gene Expression during Interspecific Hybridization
Source: PLoS One. 2015 Feb 26;10(2):e0117293. doi: 10.1371/journal.pone.0117293 (PMC4342222; doi:10.1371/journal.pone.0117293)
Supplement: S4 Table — (PDF) [file pone.0117293.s009.pdf]

TABLE S4

Expression analysis of imprinted genes in the literature

Tissue [a]	Imprinting Status [b]	Paternal Bias	# of genes	Expressed[c] Col-0 X Aa[d]	Expressed C24 X Aa	Expressed Col-0 X C24	Expressed C24 X Col-0
Endosperm (65)	Imprinted	PEG	11	7	7	9	9
	Unknown	PEG	54	51	49	47	52

[a] Seed compartment for which expression pattern was observed.
[b] TAIR annotated imprinted genes expressed in parent-of-origin specific manner with known epigenetic processes that establish allele-specific silencing. “Unknown” genes referenced in literature as having paternally-biased expression.
[c] Expressed at coverage > 5 reads.
[d] Aa, *Arabidopsis arenosa.*
