# Supplementary material for: Perturbation of Parentally Biased Gene Expression during Interspecific Hybridization
Source: PLoS One. 2015 Feb 26;10(2):e0117293. doi: 10.1371/journal.pone.0117293 (PMC4342222; doi:10.1371/journal.pone.0117293)
Supplement: S5 Table — (PDF) [file pone.0117293.s010.pdf]

TABLE S5

Unique PEGs related to cell growth and expansion

GO Category[a]	Enrichment[b]	Count[c]	Padj[d]	Gene/Protein	Function[e]
Cell wall modifications	20x	6	0.001	AT2G47040 (VANGUARD1)	pollen tube growth
				AT5G39270 (EXPANSIN22)	cell growth
				AT5G39290 (EXPANSIN26)	cell growth
				AT5G39260 (EXPANSIN21)	cell growth
				AT2G47030 (VGDH1)	silique development
				AT2G26450	silique development
Unidimensional cell growth	12x	4	ns	AT2G47040 (VANGUARD1)	pollen tube growth
				AT5G39270 (EXPANSIN22)	cell growth
				AT5G39290 (EXPANSIN26)	cell growth
				AT5G39260 (EXPANSIN1)	cell growth

[a] Gene Ontology (GO) classifications according to DAVID (http://david.abcc.ncifcrf.gov/).
[b] Prevalence relative to TAIR10 genome.
[c] Number of genes relating to particular gene class.
[d] Benjamini-Hochberg adjusted *P*-value.
[e] According to The Arabidopsis Information Resource (http://www.arabidopsis.org/).
